# Takotsubo cardiomyopathy caused by epinephrine-treated bee sting anaphylaxis: a case report

**DOI:** 10.1186/s13256-015-0722-5

**Published:** 2015-10-31

**Authors:** Diab Ghanim, Zvi Adler, Dahud Qarawani, Fabio Kusniec, Offer Amir, Shemy Carasso

**Affiliations:** 1 Department of Cardiology, B Padeh Medical Center, Poriya, Lower Galilee, Israel; 2 Department of Cardiac Surgery, Rambam Healthcare Campus, Haifa, Israel; 3 Bar Ilan University Faculty of Medicine, Zefat, Israel; 4 Noninvasive Cardiac Imaging Unit, Department of Cardiology, B Padeh Medical Center, Poriya, Lower Galilee, 15208 Israel

**Keywords:** Catecholamines, Extracorporeal membrane oxygenation (ECMO), Stress-induced cardiomyopathy

## Abstract

**Introduction:**

Stress-induced cardiomyopathy (Takotsubo) after bee stings in patients who have received catecholamines is rare. Endogenous as well as exogenous administration of catecholamines is thought to trigger stress-induced cardiomyopathy.

**Case presentation:**

A 37-year-old healthy white woman was stung by an unknown Hymenoptera that resulted in an anaphylactic reaction. Intravenous adrenaline (0.9 mg) was administered at a nearby clinic; she was transferred to our emergency room. Cardiogenic shock was diagnosed and mechanical ventilation commenced. Hemodynamic stabilization was not achieved by inotropic support and intra-aortic balloon pump insertion. Initial coronary angiography did not demonstrate any coronary obstructive lesions while her left ventricular systolic function was severely depressed. Peripheral femoral venoarterial extracorporeal membrane oxygenation was inserted as a bridge to recovery assuming possible reversible cause of the cardiogenic shock. Over the following 48 hours she was extubated and gradually weaned off venoarterial extracorporeal membrane oxygenation and inotropic support. She was discharged with a near normal left ventricular ejection fraction and in 3 weeks she was asymptomatic with normal electrocardiographic and echocardiographic examinations (left ventricular ejection fraction >65 %).

**Conclusions:**

A Hymenoptera sting may be a specific cause of catecholamine cardiac depression. The presence of cardiogenic shock and its etiology should prompt aggressive management including extracorporeal membrane oxygenation as a bridge to cardiac functional recovery in such rare scenarios.

## Introduction

There are incidental reports of stress-induced cardiomyopathy (SIC; Takotsubo or “broken heart syndrome”) after bee stings [1]. The syndrome was also reported in other anaphylaxis cases [2]. Although the treatment of choice in anaphylaxis includes acute administration of catecholamines, they may not be effective or may even be deleterious in patients with SIC because they play a major role in the pathophysiology of SIC. We present a case of a patient stung by a bee presenting with shock with an electrocardiogram (ECG) suggesting acute myocardial infarction that was unresponsive to catecholamines. Diagnostic and therapeutic challenges are discussed.

## Case presentation

**Fig. 1:**
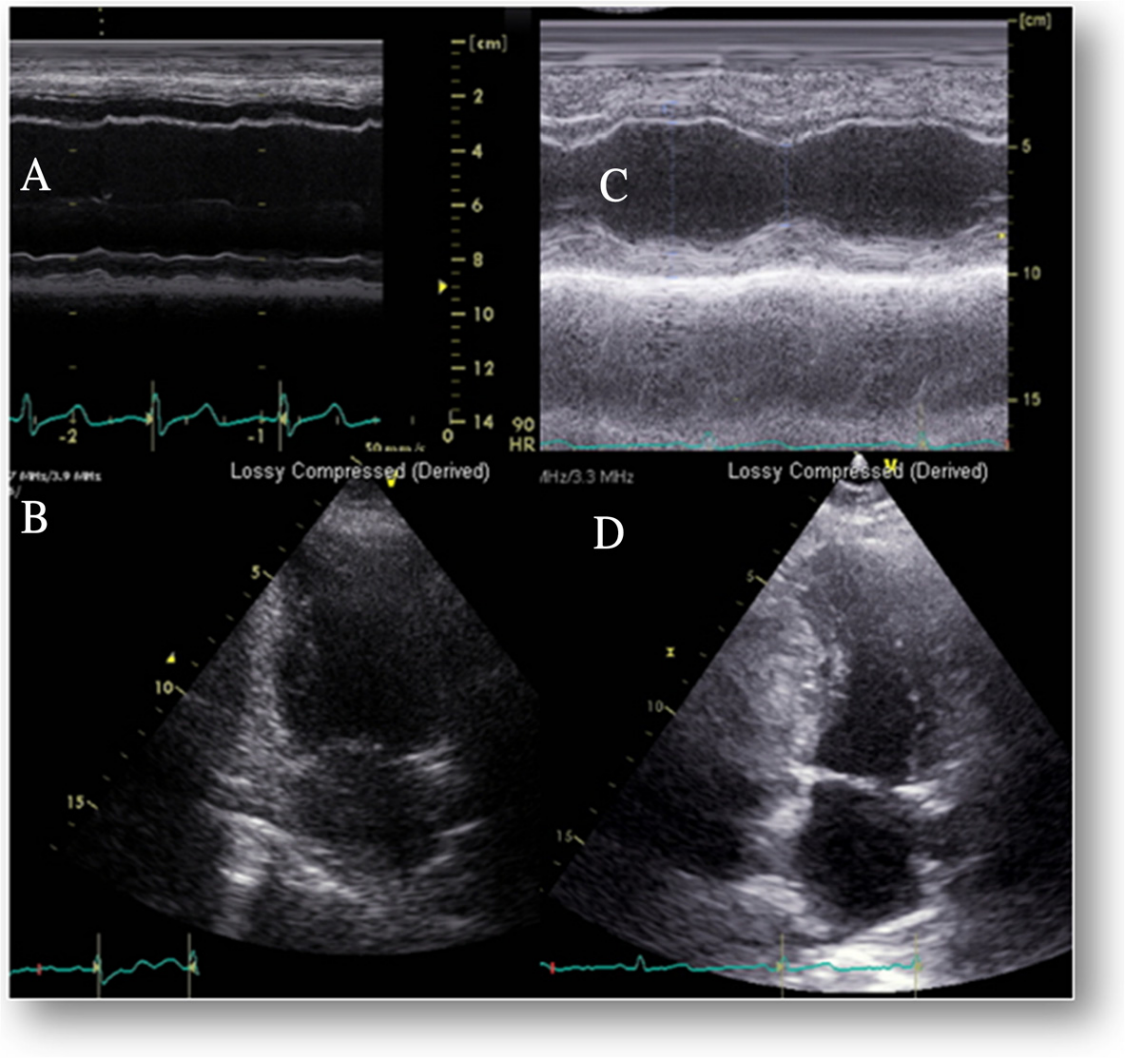
Echocardiographic findings on admission and early follow-up. **a** Admission, Basal MMode showing dilated left ventricle with severe systolic function. **b** Admission, end systolic apical 2-chambers view demonstrating large end systolic volume. **c** Follow-up at 3 weeks, Basal MMode showing normal ventricular size and contraction. **d** Follow-up at 3 weeks, end systolic apical 2-chambers view with normal ventricular size and contraction

A 37-year-old previously healthy white woman was stung by an unknown Hymenoptera; an hour later a rash developed accompanied by breathing difficulties and impaired consciousness. At a nearby clinic she was found to be hypotensive and was immediately given intravenous adrenaline (0.9 mg) and promethazine, and transferred to our emergency room. On admission she was agitated, tachypneic without stridor (approximately 24 breaths/minute), desaturated (arterial oxygen saturation, SaO_2_, approximately 84 on oxygen mask) and hypotensive (blood pressure was 85/50 mmHg) with cool and moist extremities. Her ECG demonstrated lateral wall ST elevation, inferior and anterior ST depressions. Echocardiography demonstrated left ventricular (LV) global basal and mid akinesis with preserved apical contraction, with an estimated LV ejection fraction of 20 % (Fig. 1a, b), her right ventricular cavity size was normal and its systolic function was mildly reduced. Combined cardiogenic and anaphylactic shock was therefore suggested. She was then mechanically ventilated and transferred to the cardiac catheterization laboratory for a presumed acute myocardial infarction. Cardiac catheterization revealed normal coronary angiography, severely reduced LV function with hyperdynamic apical function. The LV end diastolic pressure was 25 mmHg. At this time her blood pH was 7.2, lactate level was 3.3 mg/dl with a combined metabolic and respiratory acidosis. Her troponin I level was 0.14 ng/dl (high). An intra-aortic balloon pump was inserted and set to 1:1 augmentation, and various intravenous high-dose inotropic agents including adrenaline, norepinephrine and dopamine were administered to achieve a central systolic blood pressure of approximately 100 mmHg and appropriate arterial oxygen saturation. She remained hemodynamically unstable under this aggressive pharmacological and mechanical support. Assuming a reversible cause of cardiomyopathy, venoarterial extracorporeal membrane oxygenation (VAECMO) was installed via the femoral vessels (21 F and 18 F for venous and arterial cannulas, respectively) with limb perfusion protection. A day later, she was extubated and as her cardiac systolic function improved she was gradually weaned off VAECMO support and inotropic agents over the following 48 hours. She was discharged with a near normal LV ejection fraction and gained a full complete recovery. Three weeks after the event she was asymptomatic with normal electrocardiographic and echocardiographic examinations (LV ejection fraction >65 %, Fig. 1c, d) and she returned to her previous healthy normal life. She continued with Hymenoptera venom sensitivity testing and started a desensitization program.

## Discussion

### Hymenoptera venom adrenalin and SIC

SIC has been previously reported after bee stings and other cases of anaphylaxis [1, 2]. Common to all these reports is the high dose of adrenaline administered to patients ranging from 0.2 to 1.0 mg intravenously (IV) and up to 5.0 mg intramuscular injection (IM) similar to our patient. Endogenous catecholamines are thought to trigger SIC, but exogenous administration of various types of catecholamines has been shown to be related to its development as well [3]. All forms of SIC were found in these cases, including apical ballooning, mid or basal akinesis. Our patient demonstrated the latter form often known as “reverse Takotsubo”. Kounis syndrome [4] characterized by coronary vasospasm resulting from sudden release of histamine and other inflammatory mediators from mast cells, macrophages, and T lymphocytes leading to acute coronary syndromes may also play an important role in cardiac depression after bee stings and may even coexist with SIC [5]. Experimentally the mechanism of action with bee sting may also be catecholamine related. Wistar rats injected intravenously with Africanized bee venom showed a significant decrease in tissue noradrenalin concentration when compared to controls, suggesting tissue noradrenalin release. Although it is unclear whether the pathology was caused by the venom, anaphylactic reaction to it, or epinephrine usage, endogenous and exogenous catecholamine excess seemed to play a major part in our patient’s presentation.

### Toxic cardiomyopathy management

Extracorporeal membrane oxygenation (ECMO) is used to treat medically refractory cardiogenic shock when there is poor oxygenation, and ECMO can be a rapid option for emergency biventricular support. ECMO is a very useful method of supporting extreme cardiac failure in patients with self-terminating and fast improving conditions. Survival of patients treated with ECMO reflects the critical nature of the patients in whom it is used. Outcomes may be improved when ECMO is used for specific indications such as acute myocarditis, in which survival was reported to be as high as 75 % in adults. Our case represents a classical indication for the use of ECMO, as our patient had a severe, presumed reversible cardiac depression (either from adrenalin or bee sting-induced myocardial depression), with difficulties maintaining circulation and oxygenation by conventional methods. Complications of ECMO are common and are related to the underlining pathology needing ECMO and to the ECMO device itself (surgical insertion, circuit tubing, anticoagulation and so on) [6]. Hemorrhage, ranging between 10 and 30 % [7, 8], is the most frequent complication; it arises at the cannulation site or can be intracranial, retroperitoneal or pulmonary. Other complications include hemolysis, systemic and intracardiac thromboembolism and sepsis. VAECMO-specific complications include arterial dissections and distal ischemia.

To the best of our knowledge this is the first case of use of ECMO in anaphylaxis complicated with cardiogenic shock. ECMO was used here as a bridge to recovery emphasizing the importance of early identification of severe unresponsive cardiogenic shock and prompt application of cardiac and respiratory support before irreversible cardiac and/or end organ damage develop. Although the risks/benefits ratio should be considered before application of ECMO, it should not delay prompt insertion of the device when indicated.

## Conclusions

We present a dramatic case of Takotsubo triggered by a bee sting requiring unusual life support measures such as ECMO. It is possible that the combination of anaphylactic mediators along with endogenous and exogenous catecholamines triggers this clinical scenario, and aggressive hemodynamic support may be needed to support the patient until the effects of these mediators lapse.

## Consent

Written informed consent was obtained from the patient for publication of this case report and any accompanying images. A copy of the written consent is available for review by the Editor-in-Chief of this journal.
